# Evaluation of cerium oxide as a phosphate binder using 5/6 nephrectomy model rat

**DOI:** 10.1186/s12882-022-02904-6

**Published:** 2022-08-08

**Authors:** Akiko Hashimoto, Jiaqi Gao, Yuki Kanome, Yukihiro Ogawa, Masaharu Nakatsu, Masahiro Kohno, Koji Fukui

**Affiliations:** 1 applause Company Limited, Biko building 4F, Shinkawa 2-24-2, Chuo-ku, Tokyo, 104-0033 Japan; 2 Molecular Cell Biology Laboratory, Department of Systems Engineering and Science, Graduate School of Engineering and Science, Shibaura Institute of Technology, Fukasaku 307, Minuma-ku, Saitama, 337-8570 Japan; 3 Molecular Cell Biology Laboratory, Department of Bioscience and Engineering, College of System Engineering and Science, Shibaura Institute of Technology, Fukasaku 307, Minuma-ku, Saitama, 337-8570 Japan; 4 SIT Research Institute, Shibaura Institute of Technology, Fukasaku 307, Minuma-ku, Saitama, 337-8570 Japan

**Keywords:** Kidney, Phosphate binder, Chronic kidney disease, Cerium oxide, Hyperphosphatemia, Phosphate adsorption

## Abstract

**Background:**

The number of chronic kidney disease (CKD) patients continues to increase worldwide. CKD patients need to take phosphate binders to manage serum phosphorus concentrations. Currently, several types of phosphate binder, including lanthanum carbonate, are used. However, they each have disadvantages.

**Methods:**

In this study, we evaluated cerium oxide as a new phosphate binder *in vitro* and *in vivo*. First, cerium oxide was mixed with phosphoric acid at pH 2.5 or 7.0, and residual phosphoric acid was measured by absorption photometry using colorimetric reagent. Second, cerium oxide was fed to 5/6 nephrectomy model rats (5/6Nx), a well-known renal damage model. All rats were measured food intake, water intake, feces volume, and urine volume, and collected serum and urine were analyzed for biochemical markers.

**Results:**

Cerium oxide can adsorb phosphate at acidic and neutral pH, while lanthanum carbonate, which is a one of popular phosphate binder, does not dissolve at neutral pH. Cerium oxide-treatment reduced serum phosphate concentrations of 5/6Nx rats without an increase in serum alanine transaminase levels that would indicate hepatotoxicity, and cerium oxide-treatment maintained serum creatinine and blood urea nitrogen levels, while those of normal 5/6Nx rats increased slightly.

**Conclusions:**

These results suggest that cerium oxide can be a potential phosphate binder. Decreased body weight gain and increased water intake and urine volume in 5/6Nx rats were thought to be an effect of nephrectomy because these changes did not occur in sham operation rats. Additional investigations are needed to evaluate the longer-term safety and possible accumulation of cerium oxide in the body.

**Supplementary Information:**

The online version contains supplementary material available at 10.1186/s12882-022-02904-6.

## Background

Chronic kidney disease (CKD) is a disease in which renal function declines, and the number of CKD patients continues to increase worldwide. Progression of CKD results in end stage kidney disease (ESKD), and ESKD patients need dialysis or renal transplantation [1]. In Japan, the number of CKD patients in 2005 was estimated at 13.3 million, accounting for 12.9% of the adult population [2]. The number of dialysis patients reached 347,671 in 2020 [3], and the increase of CKD and ESKD patients has become a serious problem.

Mineral excretion is reduced in CKD patients, and decreased phosphorus excretion leads to elevated serum phosphorus levels, resulting in hyperphosphatemia. Hyperphosphatemia in CKD patients is known to be closely associated with the development of cardiovascular disease [4]. In hyperphosphatemia patients, serum phosphorus and calcium are increased, resulting in an increase in calcium-phosphate products associated with vascular calcification [4–6]. To prevent cardiovascular events, hyperphosphatemia patients should eat minimal phosphorus-containing protein and take phosphate binders. One popular phosphate binder is lanthanum carbonate (Fosrenol®) [7–13], and the others are precipitated calcium carbonate [7, 9, 10, 12, 13], sevelamer hydrochloride [12–17], bixalomer [13, 16, 17], ferric citrate hydrate [13, 18, 19], and sucroferric oxyhydroxide [13, 20, 21].

Lanthanum carbonate ionizes in gastric acid, and the ions bind to phosphate ions. Lanthanum phosphate has very low solubility and, has been reported to precipitates in the feces. Consequently, phosphate is egested without systemic absorption, and serum phosphorus levels are decreased.

However, lanthanum carbonate has two disadvantages. First, lanthanum carbonate ionizes only at low pH. The influx of stomach contents after a meal makes the stomach pH more neutral. Therefore, in clinical use, lanthanum carbonate cannot fully exert its phosphate adsorption capacity. Second, it has been reported that lanthanum depositions are observed in patients treated with lanthanum carbonate [22–25]. The effects of these depositions on living organisms have not yet been elucidated, and there are reports that lanthanum depositions are associated with cancer or inflammation [23–25]. For these reasons, a new phosphate binder is needed.

In this study, we focused on cerium oxide, which is a lanthanoid compound. Cerium oxide is used as a phosphate adsorbent and has been studied as a treatment to avoid aluminum hydroxide toxicity [26, 27]. Shibamoto et al. found that cerium oxide can be adsorbed as an anion under pH 7 by ion exchange [26]. They evaluated the phosphate adsorption capacity of cerium oxide using hyperphosphatemic dogs and column-filled cerium oxide supported on porous polymers. However, they did not orally administer cerium oxide, but used a column for passing blood. Cerium oxide as an orally administered phosphate binder has been reported by Ogura et al. in 1986 [28]. There are few or no studies of cerium oxide as an orally administered phosphate binder. In this study, we examined the effect of cerium oxide as an orally administered phosphate binder using 5/6 nephrectomy model rats (5/6Nx).

## Materials and methods

### Animals

All animal experiments were approved by the Animal Protection and Ethics Committee of Shibaura Institute of Technology (approval number #20005, permission on Aug 25^th^, 2020), which conform to the current Japanese laws. The 5/6 nephrectomy and sham-operated male Wistar rats (six weeks old) were purchased from Japan SLC, Inc. (Shizuoka, Japan). They were housed at a temperature of 22 ± 2 °C with a 12-h light–dark cycle, and *ad libitum* access to food and water. They were experimented on after a week of habituation and given Labo MR Stock (Nosan Corp., Kanagawa, Japan) during habituation.

### Materials

Lanthanum carbonate was purchased from NIKKI CORPORATION (Saitama, Japan). AIN-93G and AIN-93G (substituted with soy protein) were obtained from Funabasi Farm Co. Ltd., (Chiba, Japan). BIOMOL® Green Reagent was purchased from Enzo Life Sciences, Inc. (New York, USA). All other materials were obtained from commercially available sources unless otherwise stated.

### Phosphate adsorption test

Cerium oxide dispersion liquid (average particle size: 4 nm, 0.1 g) was added to 1.5 mL tubes, and Milli-Q water was added to make a final weight of 1 g. This was used as a 10-fold diluted dispersion liquid. The following were added to new tubes: Milli-Q water (800, 760, 740, 720, 700 μL), 500 mM glycine–HCl buffer pH 2.5 (100 μL), 10-fold diluted dispersion liquid (0, 40, 60, 80, 100 μL), and approximately 30 mM phosphoric acid solution (100 μL), resulting a final volume of 1 mL. The mixed solutions were incubated (38 °C, 1 h) and centrifuged (10,000 rpm, 5 min, room temperature (R/T)). The supernatant fraction (80 μL) was added to Milli-Q (920 μL) and mixed completely, and a part of the solution (80 μL) was added to Milli-Q (880 μL) and mixed completely. To each well of a 96-well plate, the diluted solution (50 μL) and BIOMOL® Green Reagent (100 μL) were added and incubated (R/T, 25 min). The absorbance at 620 nm was measured using a UV/Vis microplate and cuvette spectrophotometer (Multiskan GO, Thermo Fisher Scientific, Inc.; Waltham, USA). Finally, we measured the concentration of phosphoric acid solution and calculated the phosphate adsorption capacity.

This experiment was also performed using 500 mM Bis–Tris HCl pH 7.0 to evaluate the phosphate adsorption capacity at neutral pH.

### Animal experiments

**Fig. 1:**
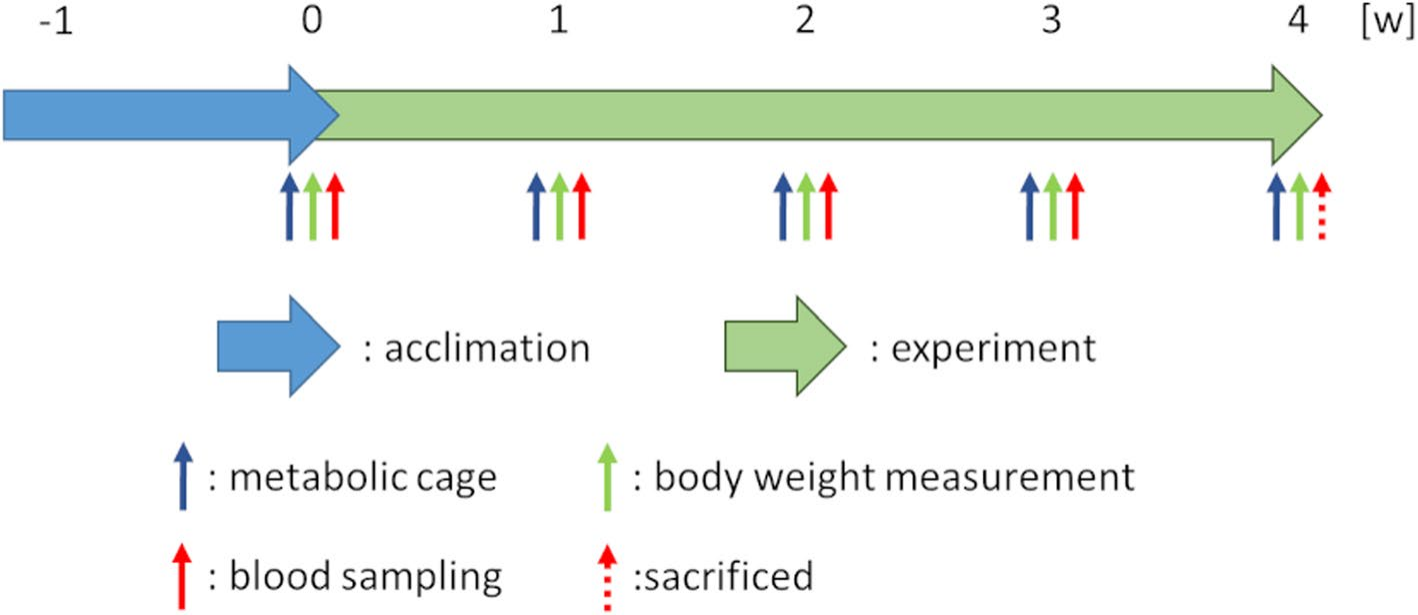
Scheme of the animal experiment

After an acclimatization period of one week, 5/6Nx or sham rats were divided by body weight into six (5/6Nx-s–n, 5/6Nx-s-La, 5/6Nx-s-Ce, 5/6Nx-c-n, 5/6Nx-c-La, or 5/6Nx-c-Ce) or two (Sham-s or Sham-c) groups (*n* = 6), respectively. During the experimental period, the rats were provided AIN-93G or AIN-93G (substituted with soy protein) as shown in Table 1. In Sham-s, Sham-c, 5/6Nx-s-n, and 5/6Nx-c-n, food was provided without the food additive. In 5/6Nx-s-La and 5/6Nx-c-La, food was provided with 0.5% lanthanum from lanthanum carbonate according to a previous report [29]. Additionally, we used 1.31% cerium oxide added to food in animal experiments due to its commensurate capacity compared to 0.5% lanthanum. In 5/6Nx-s-Ce and 5/6Nx-c-Ce, food was provided with 1.31% cerium oxide from the cerium oxide dispersion liquid. The group conditions and experimental design are indicated in Table 2 and Fig. 1, respectively. During the experimental period, the rats were placed in metabolic cages to measure food intake, water intake, feces volume, and urine volume, and urine was sampled weekly. Additionally, the rats were weighed and blood was collected from tail veins weekly. After four weeks, the rats were fasted for approximately 19 h, sacrificed, and blood was collected. The blood was stored at R/T for 1 h and centrifuged at 1400 × g for 20 min to separate serum. The serum and urine were stored at − 80 °C until biochemical analysis.

**Table 1:** Composition of base foods

		AIN-93G	AIN-93G (substituted with soy protein)
casein	%	20	-
soy protein	%	-	20
L-cystine	%	0.30	0.30
β-cornstarch	%	39.7486	39.7486
α-cornstarch	%	13.20	13.20
sucrose	%	10.00	10.00
soy oil	%	7.00	7.00
cellulose powder	%	5.00	5.00
AIN-93G mineral mix	%	3.50	3.50
AIN-93G vitamin mix	%	1.00	1.00
choline bitartrate	%	0.25	0.25
tert-butylhydroquinone	%	0.0014	0.0014

**Table 2:** Conditions of animal experimental groups

Group name	Model	Food	Food additives
Sham-s	Sham	AIN-93G (substituted with soy protein)	
Sham-c	Sham	AIN-93G	
5/6Nx-s-n	5/6 Nephrectomy	AIN-93G (substituted with soy protein)	
5/6Nx-s-La	5/6 Nephrectomy	AIN-93G (substituted with soy protein)	0.5% La from La_2_(CO_3_)_3_・12.7H_2_O
5/6Nx-s-Ce	5/6 Nephrectomy	AIN-93G (substituted with soy protein)	1.31% CeO_2_
5/6Nx-c-n	5/6 Nephrectomy	AIN-93G	
5/6Nx-c-La	5/6 Nephrectomy	AIN-93G	0.5% La from La_2_(CO_3_)_3_・12.7H_2_O
5/6Nx-c-Ce	5/6 Nephrectomy	AIN-93G	1.31% CeO_2_

### Biochemical analysis

The serum and urine were analyzed for biochemical markers including inorganic phosphate (IP), calcium (Ca), creatinine (CRE), urea nitrogen (BUN), aspartate transaminase (AST), alanine transaminase (ALT), total cholesterol (T-CHO), high density lipoprotein cholesterol (HDL-C), total protein (TP), albumin (ALB), albumin-globulin ratio (A/G), ureic acid (UA), sodium (Na), potassium (K), chlorine (Cl), amylase (AMY), triglyceride (TG), total bilirubin (T-BIL), total bile acid (TBA) and glucose (GLU) by an animal inspection service (Oriental Yeast Co., Ltd., Tokyo, Japan).

### Statistical analysis

Data were expressed as means ± SEM and analyzed with GraphPad Prism Version 9.2.0 for Windows 64-bit (GraphPad Software Inc., San Diego, USA). Comparisons between the eight groups were performed by the Tukey–Kramer method.

## Results

### Cerium oxide absorbs phosphate in a concentration-dependent manner

**Fig. 2:**
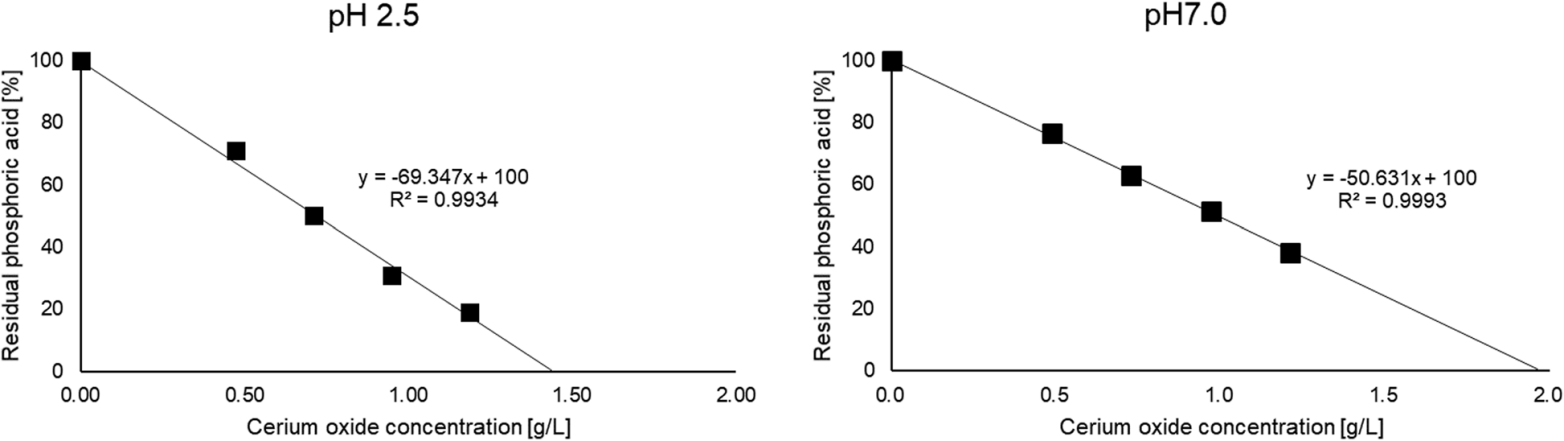
Results of the phosphate adsorption test of cerium oxide at pH 2.5 and 7.0

**Fig. 3:**
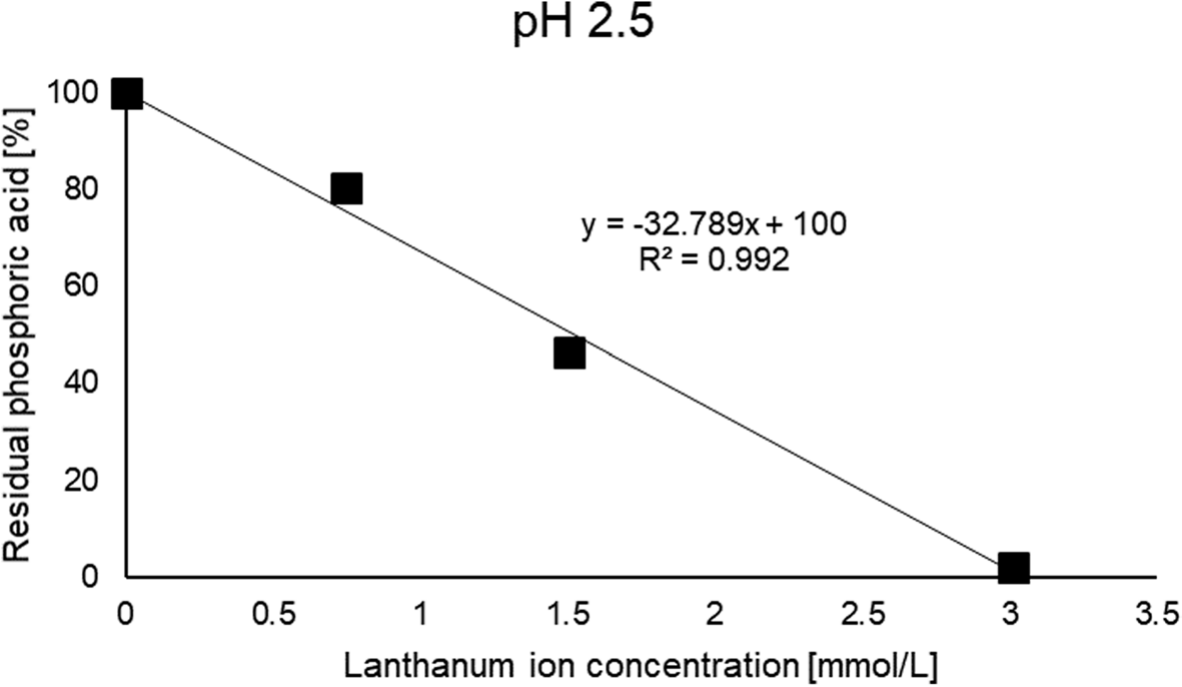
Result of the phosphate adsorption test of lanthanum carbonate at pH 2.5

The phosphate adsorption capacity of cerium oxide and lanthanum carbonate was evaluated using a colorimetric reagent (Figs. 2 and 3). Cerium oxide adsorbed phosphate in a concentration-dependent manner at pH 2.5 and 7.0. The phosphate adsorption capacity of cerium oxide at pH 2.5 was stronger than that at pH 7.0. The amount of cerium oxide necessary to adsorb phosphate at pH 7.0 was 1.4-fold that at pH 2.5. However, the decrease in phosphate adsorption capacity caused by neutral pH was not large. Lanthanum carbonate adsorbed phosphate in a concentration-dependent manner at pH 2.5, and could not be evaluated at pH 7.0 due to its poor solubility.

### Cerium oxide did not reduce the significant increase in water intake and urine volume in 5/6Nx rats

**Fig. 4:**
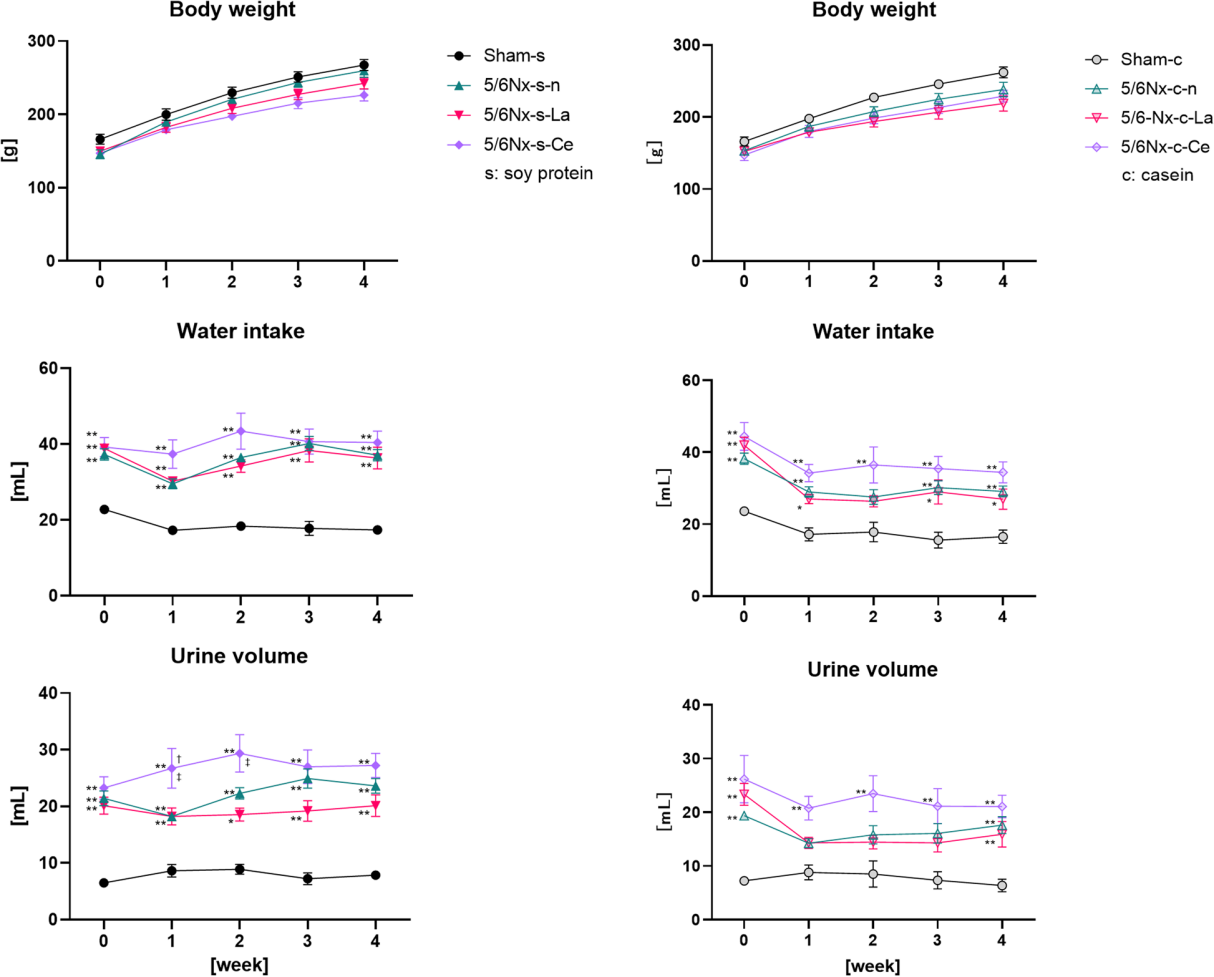
Differences in body weight, water intake, and urine volume between sham model rats and 5/6 nephrectomy model rats (5/6Nx) at each week. Sham rats and three different treated (normal, lanthanum carbonate, and cerium oxide) 5/6Nx rats fed soy protein or casein were used (Sham-s, n = 6; 5/6Nx-s–n, n = 6; 5/6Nx-s-La, n = 6; 5/6Nx-s-Ce, n = 6; Sham-c, c = 6; 5/6Nx-c-n, n = 6; 5/6Nx-c-La, n = 6; 5/6Nx-c-Ce, n = 6). The data are shown as means ± SD and were analyzed using the Tukey–Kramer method. *p < 0.05 and **p < 0.01 compared to food-matched sham rats (Sham-s or Sham-c). †*p* < 0.05 compared to food-matched normal 5/6Nx rats (5/6Nx-s–n or 5/6Nx-c-n). ‡*p* < 0.05 compared to food-matched lanthanum carbonate-treated 5/6Nx rats (5/6Nx-s-La or 5/6Nx-c-La)

The body weight, water intake, and urine volume of each rat group are shown in Fig. 4. The body weight of the 5/6Nx rats was nominally (but not significantly) lower than that of the sham rats. The body weights of lanthanum carbonate- and cerium oxide-treated 5/6Nx rats were nominally (but not significantly) lower than those of the sham rats in the presence or absence of casein in the food. There was no difference in the amount of food intake by each group. The water intake and urine volumes were significantly increased in the 5/6Nx rats compared to those of the sham rats. The both volumes of cerium oxide-treated 5/6Nx rats (5/6Nx-s-Ce and 5/6Nx-c-Ce) were nominally (but not significantly) increased compared to those of the other 5/6Nx rats. However, a significant increase in the urine volume in the 5/6Nx-s-Ce group compared to the 5/6Nx-s–n or 5/6Nx-s-La groups was only seen at one and two weeks.

### Cerium oxide significantly reduced serum IP

**Fig. 5:**
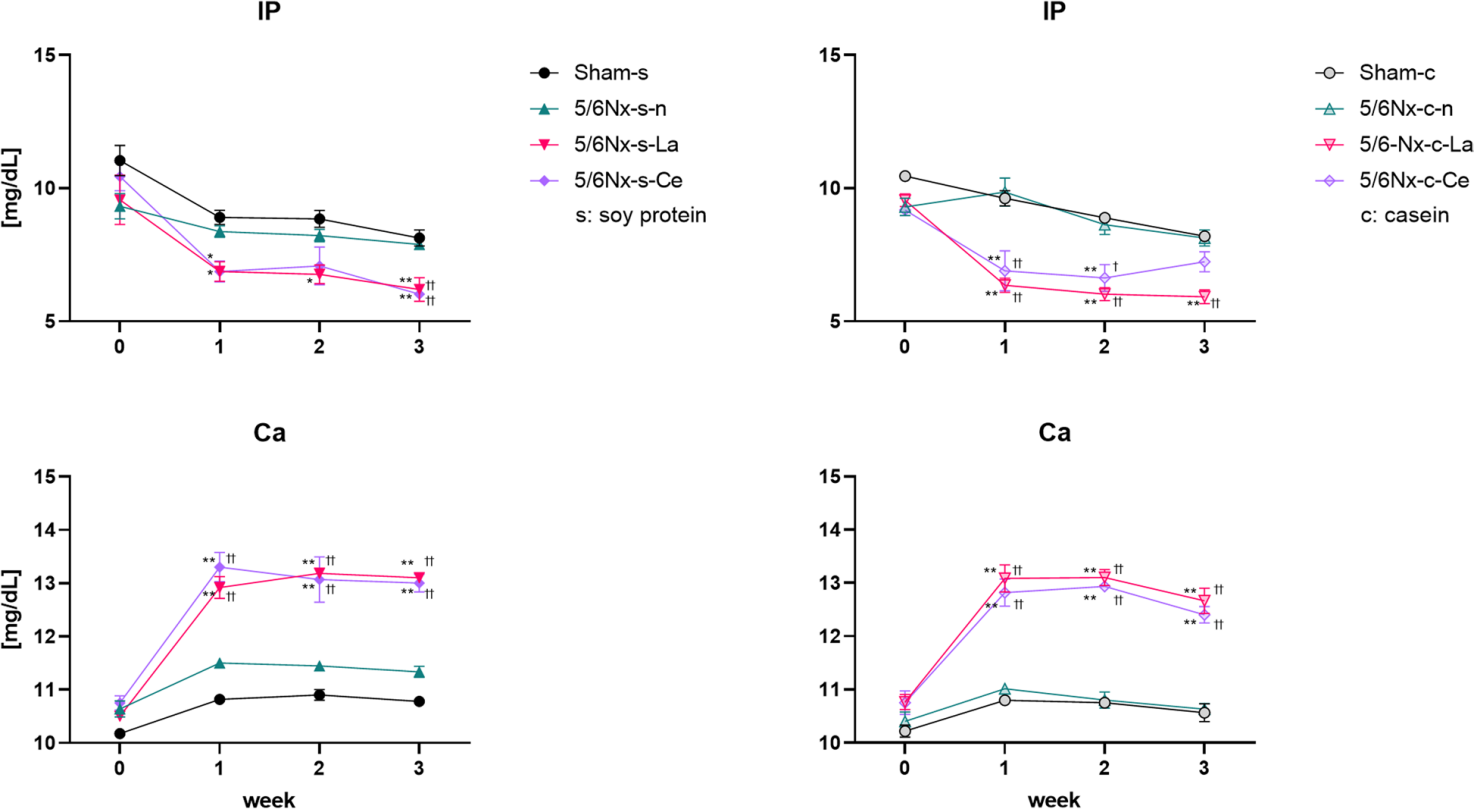
Differences in serum IP and Ca levels at each week. Sham rats and three different treated (normal, lanthanum carbonate, and cerium oxide) 5/6Nx rats fed soy protein or casein were used (Sham-s, n = 6; 5/6Nx-s–n, n = 6; 5/6Nx-s-La, n = 6; 5/6Nx-s-Ce, n = 6; Sham-c, c = 6; 5/6Nx-c-n, n = 6; 5/6Nx-c-La, n = 6; 5/6Nx-c-Ce, n = 6). The data are shown as means ± SD and were analyzed using the Tukey–Kramer method. *p < 0.05 and **p < 0.01 compared to food-matched sham rats (Sham-s or Sham-c). †*p* < 0.05 and ††*p* < 0.01 compared to food-matched normal 5/6Nx rats (5/6Nx-s–n or 5/6Nx-c-n)

Figure 5 shows the serum IP and Ca levels before and after each diet was applied for up to three weeks. The IP levels of lanthanum carbonate- and cerium oxide-treated 5/6Nx rats (5/6Nx-s-La, 5/6Nx-s-Ce, 5/6Nx-c-La, and 5/6Nx-c-Ce) were significantly lower than those of each normal diet group (Sham-s, 5/6Nx-s–n, Sham-c, and 5/6Nx-c-n). Serum Ca levels of lanthanum carbonate- or cerium oxide-treated 5/6Nx rats (5/6Nx-s-La, 5/6Nx-s-Ce, 5/6Nx-c-La, and 5/6Nx-c-Ce) were significantly higher than those of each normal diet group (Sham-s, 5/6Nx-s–n, Sham-c, and 5/6Nx-c-n). The increase of serum Ca levels occurred in parallel with the serum IP decrease.

**Fig. 6:**
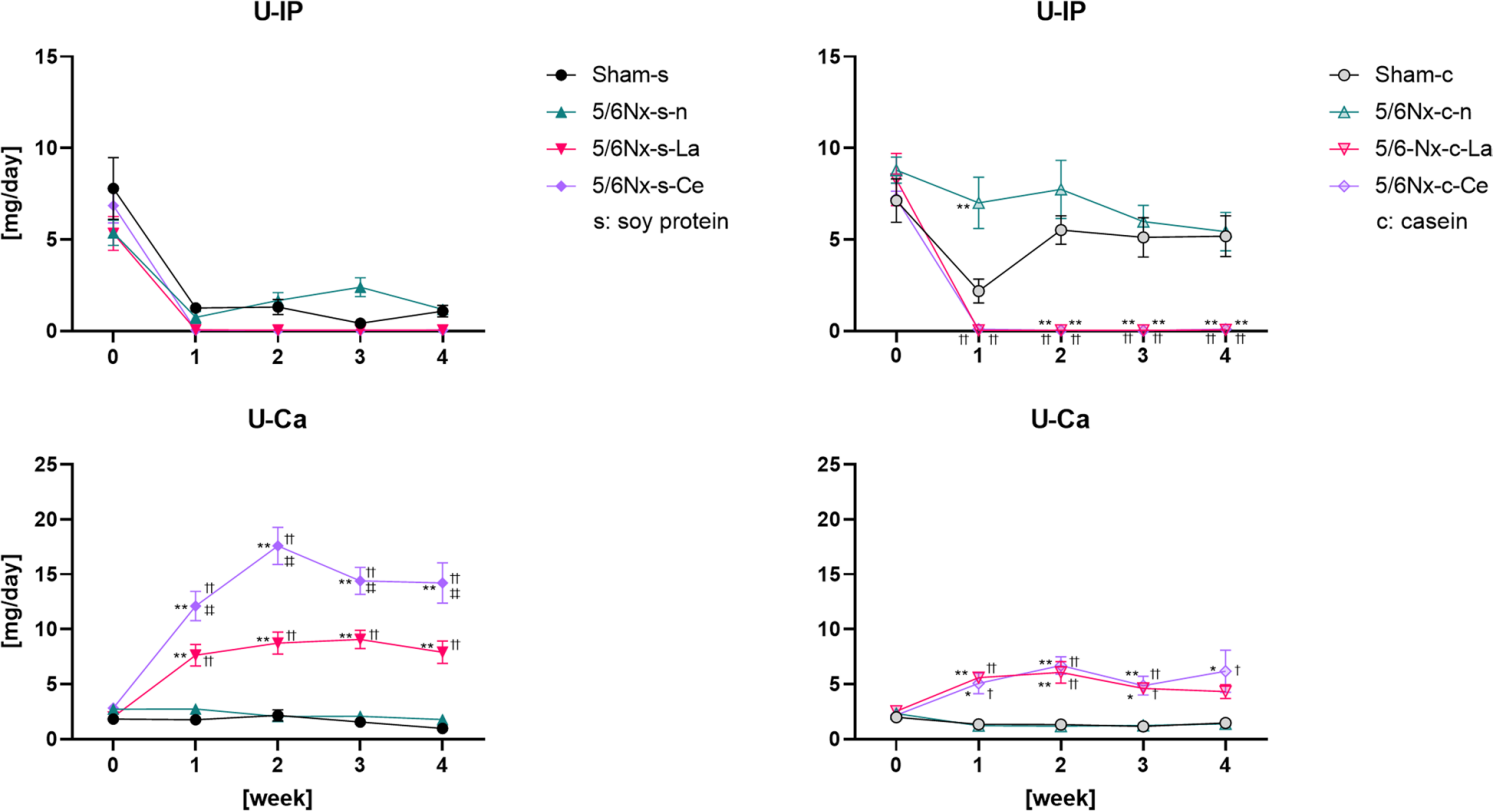
Differences in urine IP and Ca levels at each week. Sham rats and three different treated (normal, lanthanum carbonate, and cerium oxide) 5/6Nx rats fed soy protein or casein were used (Sham-s, n = 6; 5/6Nx-s–n, n = 6; 5/6Nx-s-La, n = 6; 5/6Nx-s-Ce, n = 6; Sham-c, c = 6; 5/6Nx-c-n, n = 6; 5/6Nx-c-La, n = 6; 5/6Nx-c-Ce, n = 6). The data are shown as means ± SD and were analyzed using the Tukey–Kramer method. *p < 0.05 and **p < 0.01 compared to food-matched sham rats (Sham-s or Sham-c). †*p* < 0.05 and ††*p* < 0.01 compared to food-matched normal 5/6Nx rats (5/6Nx-s–n or 5/6Nx-c-n). ‡*p* < 0.05 and ‡‡*p* < 0.01 compared to food-matched lanthanum carbonate-treated 5/6Nx rats (5/6Nx-s-La or 5/6Nx-c-La)

Figure 6 shows the urinary IP (U-IP) and Ca (U-Ca) levels before and after each diet was applied up to four weeks. The U-IP levels of lanthanum carbonate- or cerium oxide-treated 5/6Nx rats (5/6Nx-s-La, 5/6Nx-s-Ce, 5/6Nx-c-La, and 5/6Nx-c-Ce) were approximately 0 mg/day from one to four weeks. The U-Ca levels of the same four groups were significantly increased compared to those of each normal diet group (Sham-s, 5/6Nx-s–n, Sham-c, and 5/6Nx-c-n). These alterations in U-IP and U-Ca occurred in parallel with alterations of IP and Ca, respectively.

**Fig. 7:**
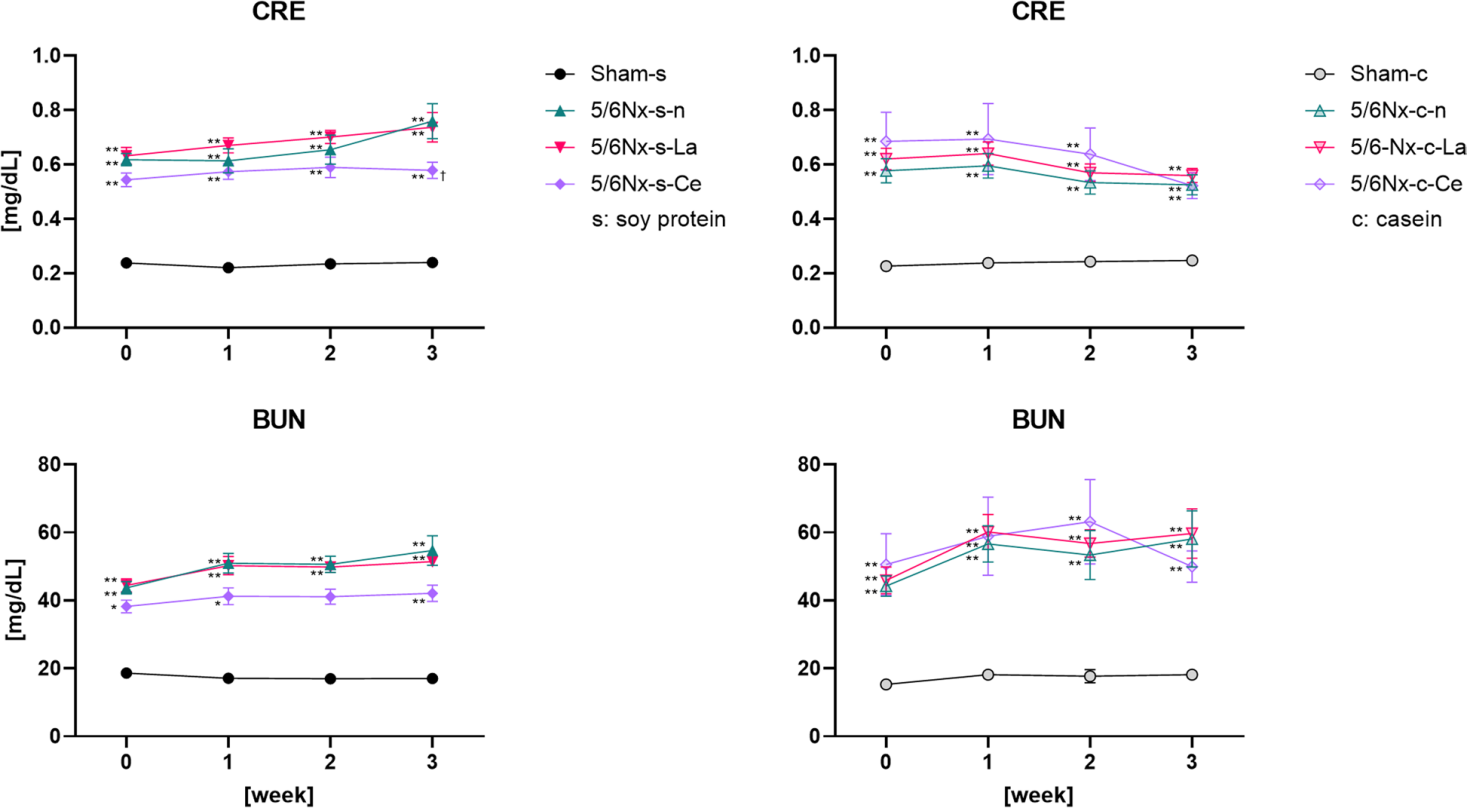
Differences in serum CRE and BUN levels at each week. Sham rats and three different treated (normal, lanthanum carbonate, and cerium oxide) 5/6Nx rats fed soy protein or casein were used (Sham-s, n = 6; 5/6Nx-s–n, n = 6; 5/6Nx-s-La, n = 6; 5/6Nx-s-Ce, n = 6; Sham-c, c = 6; 5/6Nx-c-n, n = 6; 5/6Nx-c-La, n = 6; 5/6Nx-c-Ce, n = 6). The data are shown as means ± SD and were analyzed using the Tukey–Kramer method. *p < 0.05 and **p < 0.01 compared to food-matched sham rats (Sham-s or Sham-c). †*p* < 0.05 compared to food-matched normal 5/6Nx rats (5/6Nx-s–n or 5/6Nx-c-n)

Figure 7 shows the serum CRE and BUN levels before and after each diet was applied up to three weeks. The CRE and BUN levels of the 5/6Nx rats were significantly higher than those of the sham rats. The CRE and BUN levels of these two groups (5/6Nx-s–n and 5/6Nx-s-La) were slightly increased. However, the CRE level of 5/6Nx-s-Ce did not change before the start of feeding the cerium oxide diet. The CRE and BUN levels of casein-fed cerium oxide-treated rats (5/6Nx-c-Ce) at the period from zero to two weeks were nominally (but not significantly) high compared to all other groups in the presence of casein in the food. However, their CRE and BUN levels were lower than those of casein-fed normal or lanthanum carbonate-treated rats (5/6Nx-c-n and 5/6Nx-c-La) at three weeks.

**Fig. 8:**
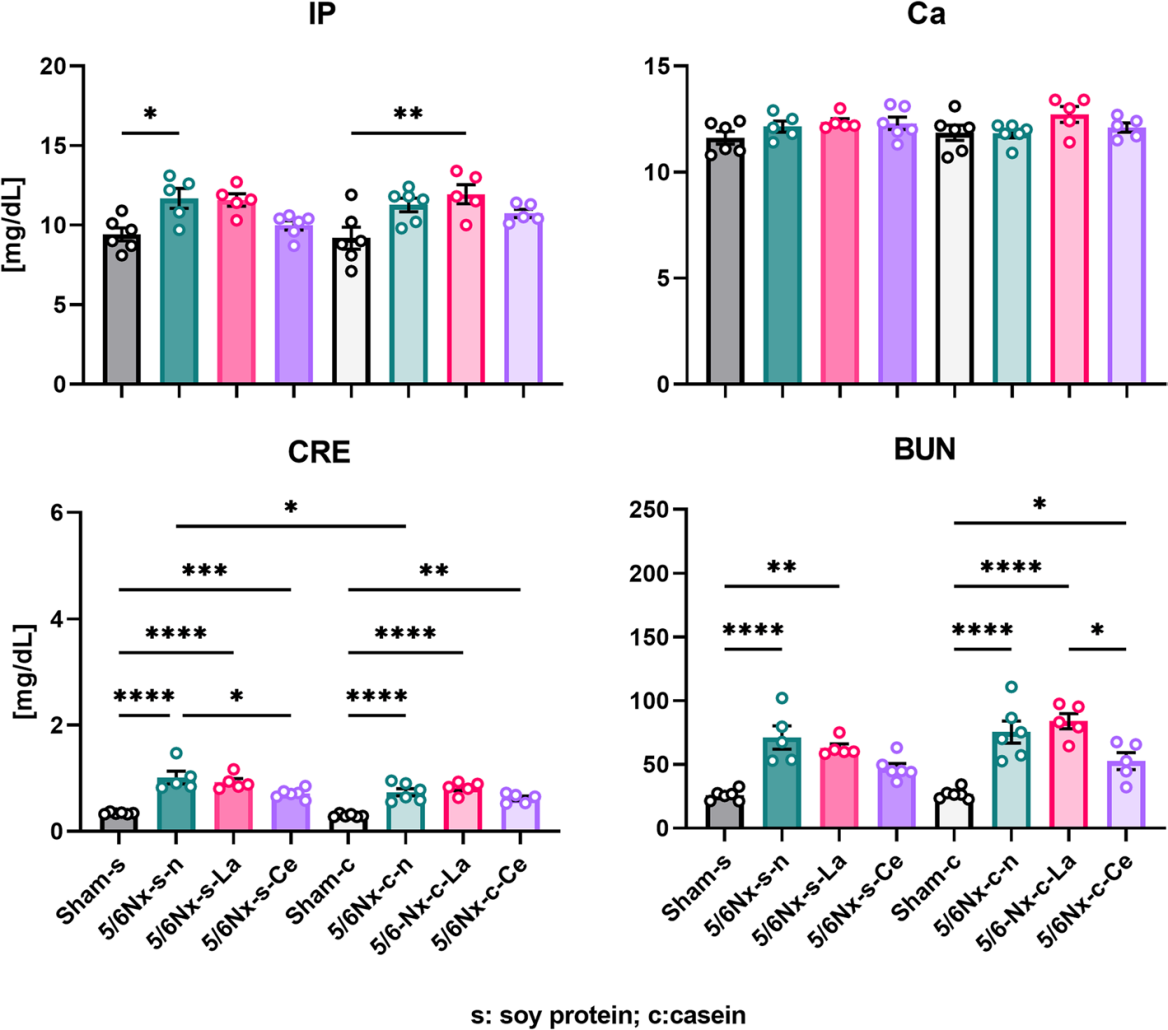
Differences in serum IP, Ca, CRE and BUN levels upon dissection. Sham rats and three different treated (normal, lanthanum carbonate, and cerium oxide) 5/6Nx rats fed soy protein or casein were used (Sham-s, n = 6; 5/6Nx-s–n, n = 6; 5/6Nx-s-La, n = 6; 5/6Nx-s-Ce, n = 6; Sham-c, c = 6; 5/6Nx-c-n, n = 6; 5/6Nx-c-La, n = 6; 5/6Nx-c-Ce, n = 5). The data are shown as means ± SD and were analyzed using the Tukey–Kramer method. *p < 0.05; **p < 0.01; ***p < 0.001; ****p < 0.0001

After the feeding period, all rats were dissected to isolate sera, and IP, Ca, CRE, and BUN levels were measured (Fig. 8). The serum IP levels of casein-fed normal or soy protein-fed lanthanum carbonate-treated 5/6Nx rats (5/6Nx-s–n and 5/6Nx-c-La) were significantly higher than those of the Sham-s and Sham-c groups, respectively. The serum Ca levels were not significantly different among any group. The CRE level of 5/6Nx rats was significantly higher than that of the sham rats. The CRE level of soy protein-fed cerium oxide-treated 5/6Nx rats (5/6Nx-s-Ce) was significantly lower than that of the 5/6Nx-s–n rats. The BUN levels of soy protein-fed normal or lanthanum carbonate-treated 5/6Nx rats (5/6Nx-s–n and 5/6Nx-s-La) were significantly higher than that of the Sham-s rats. The BUN levels of casein-fed 5/6Nx rats (5/6Nx-c-n, 5/6Nx-c-La, and 5/6Nx-c-Ce) were significantly higher than those of the Sham-c rats, and the BUN level of the 5/6Nx-c-Ce group was significantly lower than that of 5/6Nx-c-La.

### Cerium oxide did not increase the serum ALT level.

**Fig. 9:**
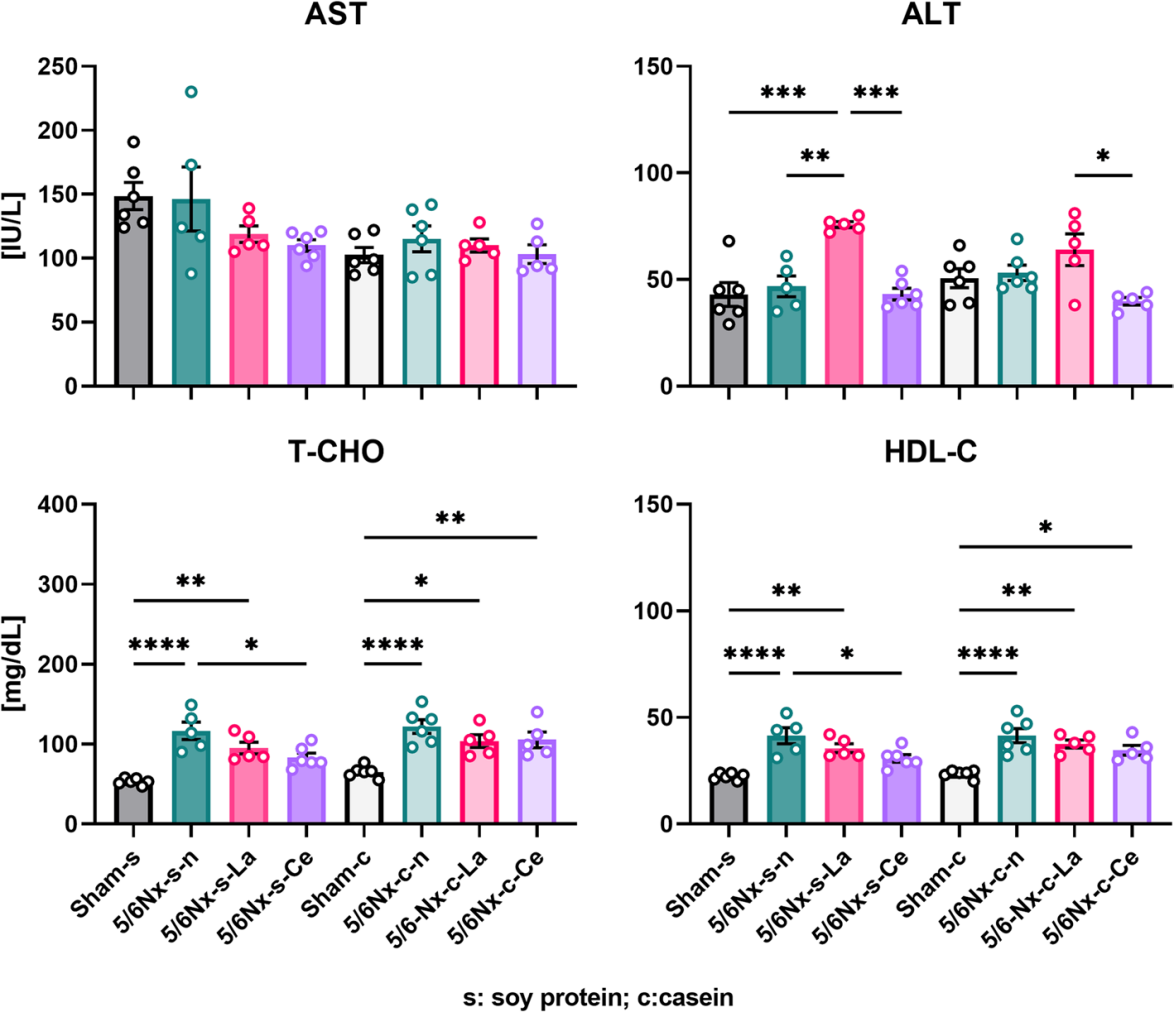
Differences in AST, ALT, T-CHO, and HDL-C levels upon dissection. Sham rats and three different treated (normal, lanthanum carbonate, and cerium oxide) 5/6Nx rats fed soy protein or casein were used (Sham-s, n = 6; 5/6Nx-s–n, n = 6; 5/6Nx-s-La, n = 6; 5/6Nx-s-Ce, n = 6; Sham-c, c = 6; 5/6Nx-c-n, n = 6; 5/6Nx-c-La, n = 6; 5/6Nx-c-Ce, n = 5). The data are shown as means ± SD and were analyzed using the Tukey–Kramer method. *p < 0.05; **p < 0.01; ***p < 0.001; ****p < 0.0001

The results of the serum parameters are shown in Fig. 9. There was no significant difference in AST level among any group. The ALT level of soy protein-fed lanthanum carbonate-treated 5/6Nx rats (5/6Nx-s-La) was significantly higher than that of the other soy protein-fed rats (Sham-s, 5/6Nx-s–n, and 5/6Nx-s-Ce). The ALT level of casein-fed lanthanum carbonate-treated 5/6Nx rats (5/6Nx-c-La) was significantly higher than that of the casein fed cerium oxide-treated 5/6Nx rats (5/6Nx-c-Ce). The T-CHO levels of the 5/6Nx rat groups were significantly higher than those of each sham group, except for soy protein-fed cerium oxide-treated 5/6Nx rats (5/6Nx-s-Ce). Additionally, the T-CHO of soy protein-fed cerium oxide-treated 5/6Nx rats (5/6Nx-s-Ce) was significantly lower than that of the soy protein-fed normal 5/6Nx rats (5/6Nx-s–n). The HDL-C levels of the 5/6Nx rat groups were higher than those of the sham rat groups, except for the soy protein-fed cerium oxide-treated 5/6Nx rats (5/6Nx-s-Ce), which showed no significant difference compared to the Sham-s group. The soy protein-fed cerium oxide-treated 5/6Nx rats (5/6Nx-s-Ce) had similarly lower values of HDL-C than the soy protein-fed normal 5/6Nx rats (5/6Nx-s–n).

Other serum indexes are shown in additional file 1 (Fig. 10). The TP levels of cerium oxide-treated 5/6Nx rats (5/6Nx-s-Ce and 5/6Nx-c-Ce) were significantly lower than those of the Sham-s and Sham-c rats, respectively. The casein-fed normal 5/6Nx rats (5/6Nx-c-n) showed similarly low TP levels compared to the Sham-c rats. The ALB levels of the 5/6Nx rat groups were significantly lower than those of the sham rat groups, except for the soy protein-fed lanthanum carbonate-treated 5/6Nx rats (5/6Nx-s-La). However, treatment with lanthanum carbonate did not improve serum ALB levels. The A/G level of soy protein-fed normal 5/6Nx rats (5/6Nx-s–n) was significantly lower than those of the other soy protein fed rats (Sham-s, 5/6Nx-s-La, and 5/6Nx-s-Ce). The casein-fed normal 5/6Nx rats (5/6Nx-c-n) showed significantly lower A/G level compared to the Sham-c rats. The Cl levels of cerium oxide-treated 5/6Nx rats (5/6Nx-s-Ce and 5/6Nx-c-Ce) were significantly different compared to each normal diet group (Sham-s, 5/6Nx-s–n, Sham-c, and 5/6Nx-c-n). The other serum indexes including UA, Na, K, AMY, TG, T-BIL, TBA, and GLU were not significantly different among any groups.

## Discussion

First, phosphate adsorption capacity of cerium oxide was evaluated *in vitro*. Cerium oxide showed phosphate adsorption at not only acidic pH but also neutral pH (Fig. 2). This is advantageous for a phosphate binder due to the increase in gastric pH after eating. The phosphate adsorption capacity of lanthanum carbonate was evaluated by a similar method (Fig. 3). The trivalent cation, Lanthanum ion binds to the trivalent anion, phosphate ion, at a ratio of 1 to 1 in an ideal environment. Our experiments indicated almost a theoretical phosphate adsorption capacity of lanthanum carbonate at pH 2.5. However, lanthanum carbonate could not be evaluated at pH 7.0 due to its poor solubility. These results indicate that cerium oxide may be a better phosphate binder than lanthanum carbonate.

Second, we evaluated cerium oxide as a potential of phosphate binder when orally administered *in vivo*. That the body weights of 5/6Nx rats were lower than those of sham rats (Fig. 4). It has been reported elsewhere that 5/6Nx rats have low body weights compared to sham rats [30–32], which is thought to be an effect of nephrectomy. Additionally, lanthanum carbonate- or cerium oxide-treated groups had suppressed body weight gain (Fig. 4). Yoshida et al. reported that lanthanum carbonate-treated rats had slightly suppressed body weight gain [29]. They found that the cause of suppressed body weight gain was the indirect effect of low IP conditions as opposed to the direct action of lanthanum. In this study, inhibited body weight gain not only occurred in lanthanum carbonate-treated 5/6Nx rats (5/6Nx-s-La and 5/6Nx-c-La), but also in cerium oxide-treated 5/6Nx rats (5/6Nx-s-Ce and 5/6Nx-c-Ce). Therefore, the suppression of body weight gain is an effect of low IP conditions.

Water intake and urine volumes of 5/6Nx rats were increased compared to the sham rats (Fig. 4). Barata et al. and Liu et al. reported similar results in which water intake and urine volumes in 5/6Nx rats were larger than in sham rats [31, 32], which is thought to be an effect of nephrectomy. Water intake and urine volumes of the cerium oxide-treated 5/6Nx rats (5/6Nx-s-Ce and 5/6Nx-c-Ce) were slightly larger than those of the other 5/6Nx rat groups, which is apparently an effect of eating a diet that includes the cerium oxide dispersion liquid. Urine volume was changed in concert with water intake volume in each group. These results suggests that water balance in the body was maintained during cerium oxide treatment.

The four groups of lanthanum carbonate- or cerium oxide-treated 5/6Nx rats (5/6Nx-s-La, 5/6Nx-s-Ce, 5/6Nx-c-La, and 5/6Nx-c-Ce) had low IP and U-IP levels compared to each normal diet group (Sham-s, 5/6Nx-s–n, Sham-c, and 5/6Nx-c-n) (Figs. 5 and 6). This suggests that 1.31% cerium oxide had a phosphate adsorption capacity comparable to 0.5% lanthanum from lanthanum carbonate. Cerium oxide probably adsorbed phosphate in the stomach and small intestine, resulting in inhibition of phosphate intake. As a result, low IP and U-IP levels in cerium oxide-treated 5/6Nx rats (5/6Nx-s-Ce and 5/6Nx-c-Ce) were observed. In this study, 5/6Nx rats did not have hyperphosphatemia, because the IP levels were equal between normal 5/6Nx rats (5/6Nx-s–n and 5/6Nx-c-n) and sham rats (Sham-s and Sham-c). Lanthanum carbonate- and cerium oxide-treated 5/6Nx rats (5/6Nx-s-La, 5/6Nx-s-Ce, 5/6Nx-c-La, and 5/6Nx-c-Ce) had low IP levels compared to each normal diet group (Sham-s, 5/6Nx-s–n, Sham-c, and 5/6Nx-c-n), which implies normal IP levels. The lanthanum carbonate- or cerium oxide-treated 5/6Nx rats (5/6 N-s-La, 5/6Nx-s-Ce, 5/6Nx-c-La, and 5/6Nx-c-Ce) had high Ca and U-Ca levels compared to each normal diet group (Sham-s, 5/6Nx-s–n, Sham-c, and 5/6Nx-c-n; Figs. 5 and 6). These variations appeared negatively correlated with variations in IP and U-IP. Yoshida et al. reported that Ca is increased by lanthanum carbonate treatment [29]. They discussed that this phenomenon is caused by low IP levels, resulting in deossification or diminishing calcium intake inhibition by phosphoric acid. Additionally, Ben-Dov et al. reported similar results in which the Ca levels of lanthanum carbonate-treated rats were increased inversely with the IP level [33].

The IP and Ca levels at dissection are shown in Fig. 8. The IP levels of lanthanum carbonate- or cerium oxide-treated 5/6Nx rats (5/6Nx-s-La, 5/6Nx-s-Ce, 5/6Nx-c-La, and 5/6Nx-c-Ce) were not as low but were comparable to normal 5/6Nx rats (5/6Nx-s–n and 5/6Nx-c-n). The Ca levels of lanthanum carbonate- or cerium oxide-treated 5/6Nx rats (5/6Nx-s-La, 5/6Nx-s-Ce, 5/6Nx-c-La, and 5/6Nx-c-Ce) were not high compared to each normal diet group (Sham-s, 5/6Nx-s–n, Sham-c, and 5/6Nx-c-n). These data indicated a different trend with that found at 1–3 weeks, which may be an effect of fasting before sacrifice. The variations in IP and Ca levels in lanthanum carbonate- or cerium oxide-treated 5/6Nx rats (5/6Nx-s-La, 5/6Nx-s-Ce, 5/6Nx-c-La, and 5/6Nx-c-Ce) were due to the effects of each treatment. Therefore, the IP and Ca levels after fasting were comparable in all 5/6Nx rat groups.

The CRE and BUN levels of 5/6Nx rats were significantly higher than those of the sham rats (Figs. 7 and 8). These results are consistent with previous reports [30–32] and indicated a decline in renal function. The CRE and BUN levels of normal or lanthanum carbonate-treated 5/6Nx rats (5/6Nx-s–n, 5/6Nx-s-La, 5/6Nx-c-n, and 5/6Nx-c-La) slowly increased. The CRE and BUN levels of cerium oxide-treated 5/6Nx rats (5/6Nx-s-Ce and 5/6Nx-c-Ce) were maintained or decreased. In 5/6Nx-s-Ce, the CRE and BUN levels were slightly lower at the start. Therefore, it is less certain that cerium oxide affects the CRE and BUN levels. However, cerium oxide conceivably had a protective effect on renal function. Nemoto et al. reported that 5/6Nx rats treated with sucroferric oxyhydroxide, which binds with phosphate, attenuated glomerulosclerosis and tubulointerstitial injury [34]. Additionally, it was reported that phosphate loading induced a decline in renal function [35]. In our study, we used two phosphate binders, namely lanthanum carbonate and cerium oxide, and each treated group showed commensurate IP levels. However, lanthanum carbonate-treated groups (5/6Nx-s-La and 5/6Nx-c-La) did not show either maintenance or decrease of the CRE and BUN levels. We believe that the mechanism of the renal protective effect of cerium oxide is unrelated to the decline in phosphate loading.

Figure 9 shows the serum AST and ALT levels, which are biomarkers of liver function. The ALT levels of lanthanum carbonate-treated 5/6Nx rats (5/6Nx-s-La and 5/6Nx-c-La) were high compared to the other groups, although the AST levels were comparable. There are many reports that lanthanum carbonate does not increase ALT [33, 36, 37]. However, the drug package insert of Fosrenol®, which includes lanthanum carbonate, describes ALT increase as a side effect that occurs in less than 1% of patients. Lanthanum carbonate possibly has weak hepatic toxicity, though cerium oxide did not increase ALT and AST. This suggests that cerium oxide is a better potential drug than lanthanum carbonate.

The serum T-CHO and HDL-C levels are shown in Fig. 9. The T-CHO and HDL-C levels in normal 5/6Nx rats (5/6Nx-s–n and 5/6Nx-c-n) were high compared to sham rats (Sham-s and Sham-c). In general, it is well known that hyperlipidemia causes CKD. The 5/6Nx rats were had a decline renal function by nephrectomy, and as a result, the T-CHO and HDL-C levels in 5/6Nx rats were increased. Kasiske et al. reported that there was an increase of cholesterol in 5/6Nx rats [38]. It is striking that the T-CHO and HDL-C levels of soy protein-fed cerium oxide-treated 5/6Nx rats (5/6Nx-s-Ce) were significantly lower than in soy protein-fed normal 5/6Nx rats (5/6Nx-s–n). These results are conceivably related to the CRE and BUN levels in cerium oxide-treated 5/6Nx rats (5/6Nx-s-Ce) that suggested a protective effect on renal function.

Additional file 1 shows the serum TP, ALB, A/G, UA, Na, K, Cl, AMY, TG, T-BIL, TBA, and GLU levels. The TP and ALB levels of cerium oxide-treated 5/6Nx rats (5/6Nx-s-Ce and 5/6Nx-c-Ce) were significantly lower than those of the sham rats (Sham-s and Sham-c). The differences were very slight, and the A/G level was not significantly different. For this reason, we do not believe these differences to be important. The Cl levels of cerium oxide-treated groups (5/6Nx-s-Ce and 5/6Nx-c-Ce) were significantly higher than those of each normal diet group (Sham-s, 5/6Nx-s–n, Sham-c, and 5/6Nx-c-n). The chloride ions are believed to be derived from the cerium oxide dispersion liquid that was used in this study; the cerium oxide-treated 5/6Nx rats (5/6Nx-s-Ce and 5/6Nx-c-Ce) consumed chloride ions in their diet, resulting in an increase in Cl levels. The other serum indexes did not show any differences.

In this study, we used the food of casein presence or absence. Casein has cation adsorption capacity [39]. However, all results were not clearly influenced by protein source (*i.e.*, casein or soy protein).

We not researched biological distribution of cerium oxide. Kumari et al. reported that cerium oxide was absorbed in body at orally administration [40]. They orally administered cerium oxide nanoparticles (30, 300, 600 mg/kg bw/day) to rats for 28 days. As a result, cerium detected in liver, kidney, brain, spleen, heart and blood. High dose (600 mg/kg bw/day) group was 4–10 μg/g in these organs. These results suggest that orally administered cerium oxide is retained in some organs. Thus, the bioaccumulation and safety of the long-term use of cerium oxide have to be evaluated in future research.

## Conclusion

In this study, we found that cerium oxide is a potential orally administered phosphate binder. We evaluated cerium oxide as a phosphate adsorption reagent *in vitro* and *in vivo.* Our results indicated that cerium oxide has phosphate adsorption capacity at acidic and neutral pH *in vitro*. The animal experiment using 5/6Nx rats suggested that cerium oxide can decrease serum IP levels similarly to lanthanum carbonate without hepatotoxicity. In addition, cerium oxide-treated 5/6Nx rats showed slightly lowered levels of CRE and BUN, which suggested a potential protective effect on renal function. Therefore, cerium oxide is a capability of phosphate binder. However, it is necessary to evaluate its protective effect on renal function with further research.

## Supplementary Information

**Additional file 1: Fig. 10.** Concentrations of biomarkers upon dissection. Sham rats and three different treated (normal, lanthanum carbonate, and cerium oxide) 5/6Nx rats fed soy protein or casein were used (Sham-s, n=6; 5/6Nx-s-n, n=6; 5/6Nx-s-La, n=6; 5/6Nx-s-Ce, n=6; Sham-c, c=6; 5/6Nx-c-n, n=6; 5/6Nx-c-La, n=6; 5/6Nx-c-Ce, n=5). The data are shown as means ± SD and were analyzed using the Tukey-Kramer method. *p<0.05; **p<0.01; ***p<0.001; ****p<0.0001

## Data Availability

All data generated or analysed during this study are included in this published article.

## Abbreviations

5/6Nx rats: 5/6 Nephrectomy model rats
CKD: Chronic kidney disease
ESKD: End stage kidney disease
IP: Serum inorganic phosphate
Ca: Serum calcium
CRE: Serum creatinine
BUN: Serum urea nitrogen
AST: Aspartate transaminase
ALT: Alanine transaminase
T-CHO: Total cholesterol
HDL-C: High density lipoprotein cholesterol
TP: Serum total protein
ALB: Serum albumin
A/G: Albumin-globulin ratio
UA: Serum ureic acid
Na: Serum sodium
K: Serum potassium
Cl: Serum chlorine
AMY: Serum amylase
TG: Triglyceride
T-BIL: Serum total bilirubin
TBA: Total bile acid
GLU: Serum glucose
U-IP: Urinary inorganic phosphate
U-Ca: Urinary calcium
