# Supplementary material for: Evaluation of cerium oxide as a phosphate binder using 5/6 nephrectomy model rat
Source: BMC Nephrol. 2022 Aug 8;23:277. doi: 10.1186/s12882-022-02904-6 (PMC9358871; doi:10.1186/s12882-022-02904-6)
Supplement: Supplementary file 1 — Additional file 1: Fig. 10. Concentrations of biomarkers upon dissection. Sham rats and three different treated (normal, lanthanum carbonate, and cerium oxide) 5/6Nx rats fed soy protein or casein were used (Sham-s, n=6; 5/6Nx-s-n, n=6; 5/6Nx-s-La, n=6; 5/6Nx-s-Ce, n=6; Sham-c, c=6; 5/6Nx-c-n, n=6; 5/6Nx-c-La, n=6; 5/6Nx-c-Ce, n=5). The data are shown as means ± SD and were analyzed using the Tukey-Kramer method. *p<0.05; **p<0.01; ***p<0.001; ****p<0.0001 [file 12882_2022_2904_MOESM1_ESM.docx]

Additional file 1


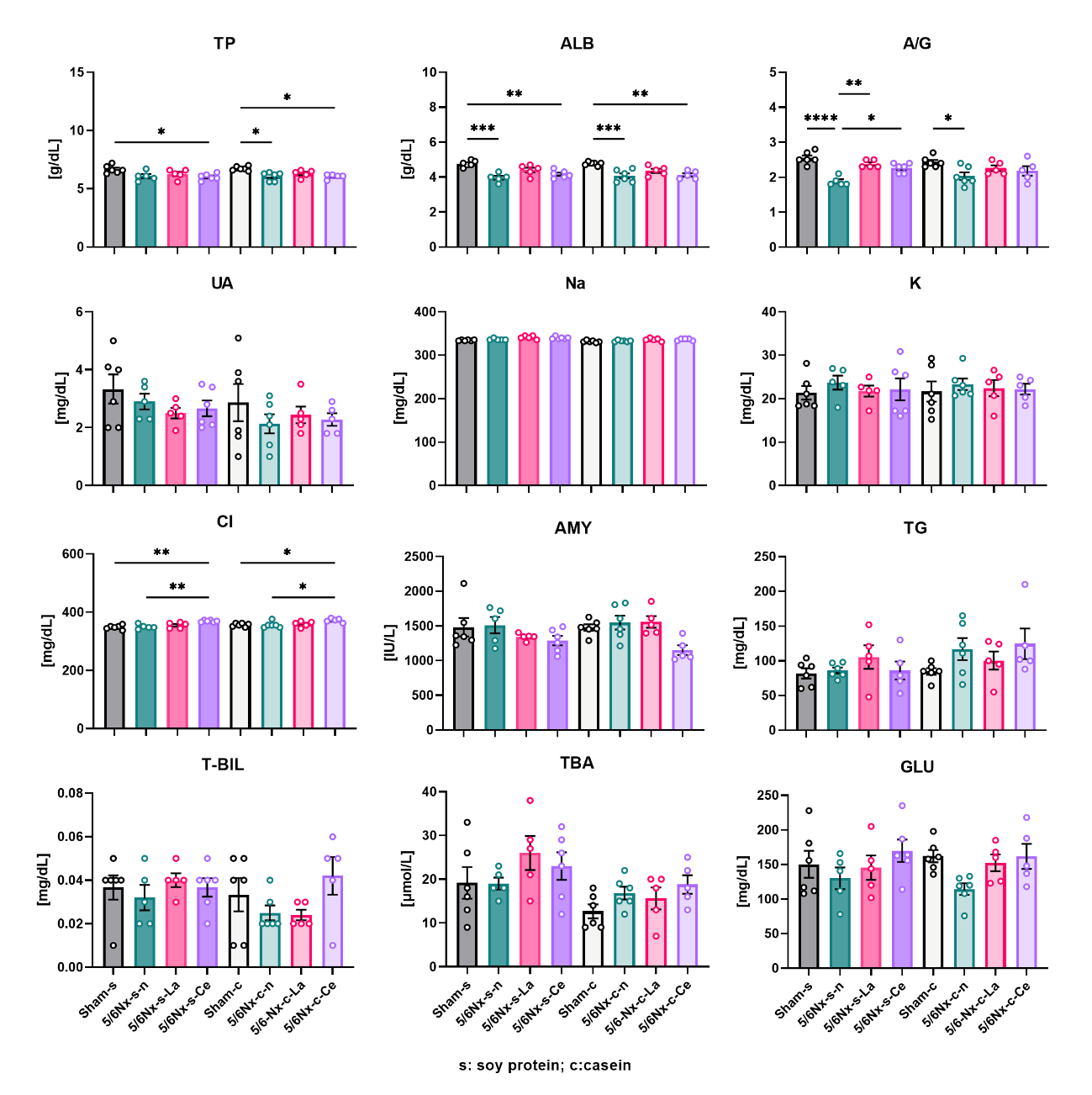


Fig. 10. Concentrations of biomarkers upon dissection. Sham rats and three different treated (normal, lanthanum carbonate, and cerium oxide) 5/6Nx rats fed soy protein or casein were used (Sham-s, n=6; 5/6Nx-s-n, n=6; 5/6Nx-s-La, n=6; 5/6Nx-s-Ce, n=6; Sham-c, c=6; 5/6Nx-c-n, n=6; 5/6Nx-c-La, n=6; 5/6Nx-c-Ce, n=5). The data are shown as means ± SD and were analyzed using the Tukey-Kramer method. *p<0.05; **p<0.01; ***p<0.001; ****p<0.0001.
